# Impact of Monomer Selection and Behavior on Transdermal Drug Delivery Systems: Regulatory and Formulation Perspectives

**DOI:** 10.7759/cureus.104819

**Published:** 2026-03-07

**Authors:** Piyush Modi, Jigneshkumar Modasiya, Dhaval Desai

**Affiliations:** 1 Regulatory Affairs, Amneal Pharmaceuticals LLC, New York, USA; 2 Regulatory Affairs, Zydus Pharmaceuticals (USA) Inc., Pennington, USA

**Keywords:** acrylic and methacrylate adhesives, critical quality attributes (cqas), ema/fda guidance, extractables and leachables (e&l), monomers, polymer chemistry, polymer-drug interactions, regulatory expectations, residual monomers, transdermal drug delivery system (tdds)

## Abstract

This review article provides a comprehensive overview of the main monomers used in transdermal drug delivery systems (TDDS), such as acrylates, methacrylates, polyethylene glycol acrylates, crosslinking monomers, and specialty monomers. It examines their impact on key quality attributes (critical quality attributes), including drug release kinetics, adhesion-cohesion balance, mechanical strength, and stability under thermal and humidity stress. The article also summarizes Food and Drug Administration and European Medicines Agency requirements for monomer characterization in regulatory submissions, details analytical techniques for measuring residual monomers and analyzing the resulting polymers, and highlights recent developments in the regulatory landscape. Monomer selection is emphasized as a vital scientific and regulatory factor that influences TDDS quality, performance, and patient safety.

## Introduction and background

Overview of transdermal drug delivery systems

**Figure 1:**
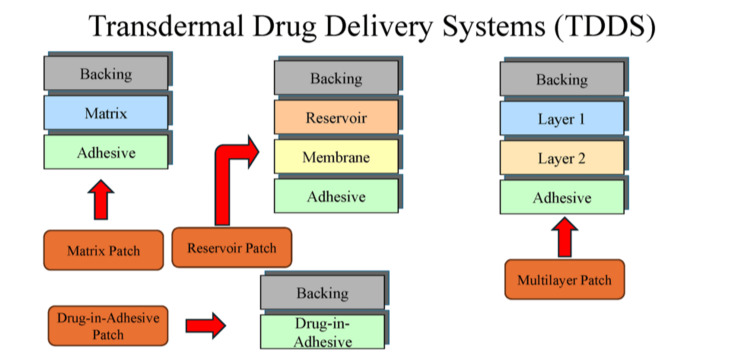
Overview of transdermal drug delivery system components. Image credit: Created by the authors using Microsoft PowerPoint (Microsoft Corporation, Redmond, WA, US).

Transdermal drug delivery systems (TDDS) administer therapeutic agents through the skin to produce either systemic or localized effects. This approach maintains relatively stable plasma concentrations, reduces dosing frequency, and improves patient compliance with treatment regimens [1]. The stratum corneum, the outermost layer of the skin, serves as the main barrier to drug absorption. TDDS are designed to surpass this barrier by modifying formulation properties and utilizing advanced delivery technologies [2]. A typical patch includes a drug reservoir or matrix, an adhesive layer for skin contact, a protective backing layer, and a release liner that is removed before application. Common configurations include matrix systems, reservoir systems, drug-in-adhesive designs, and multilaminate structures, each offering distinct release profiles and manufacturing or user benefits [3]. Figure 1 presents the structural layers of TDDS and outlines the main patch types, including matrix, reservoir, drug‑in‑adhesive, and multilayer designs for controlled release.

Role of polymeric matrices in transdermal drug delivery systems

**Figure 2:**
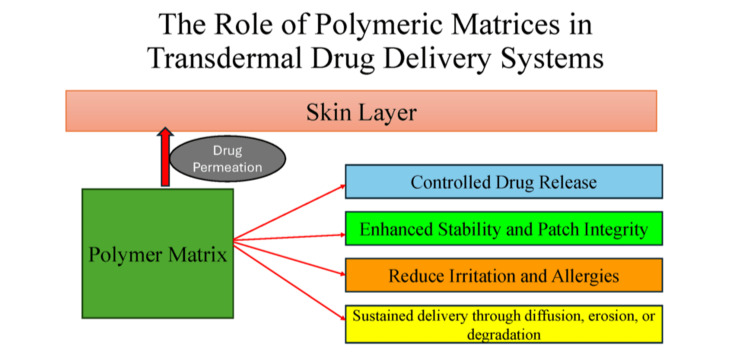
Schematic representation of polymeric matrices in transdermal drug delivery systems. Image credit: Created by the authors using Microsoft PowerPoint (Microsoft Corporation, Redmond, WA, US).

Polymeric matrices, which are networked polymer structures, provide both mechanical support and functional performance in transdermal delivery systems. In matrix-based designs, the drug is uniformly dispersed within the polymer, and the polymer’s physicochemical properties control drug transport to the skin surface. The selection of natural, semisynthetic, and synthetic polymers allows precise modulation of drug release rates and can improve patient tolerability [4]. Appropriate polymer selection is critical, as it determines the system’s critical quality attributes (CQAs). CQAs include adhesion, retention, drug delivery efficiency, stability, and overall therapeutic performance [5]. Figure 2 illustrates how polymeric matrices control drug release, support patch stability, reduce irritation, and enable sustained delivery through diffusion, erosion, or degradation.

## Review

Monomers in transdermal systems

Acrylate and Methacrylate Monomers (Foundation of Pressure-Sensitive Adhesive Systems)

Acrylates and methacrylates are widely used in TDDS adhesives for their tunable adhesion, flexibility, and compatibility with a range of active pharmaceutical ingredients (APIs). Among acrylates, 2‑ethylhexyl acrylate (2‑EHA) imparts softness, flexibility, and tack, while butyl acrylate (BA) increases flexibility and adhesive performance, and ethyl acrylate (EA) is frequently employed to adjust glass transition temperature and cohesive strength [6,7]. Acrylic acid (AA) is often added in small amounts to increase polarity and hydrogen-bonding capacity, thereby enhancing cohesion and, in some cases, improving drug solubility; however, higher concentrations may increase the risk of skin irritation [6]. Among methacrylates, methyl methacrylate (MMA) contributes rigidity and film integrity, and hydroxyethyl methacrylate (HEMA) introduces hydrophilicity that can improve compatibility with hydrophilic drugs [7]. Functional acrylates, such as hydroxyethyl acrylate (HEA) and pyrrolidonoethyl acrylate (PyEA), can further enhance drug solubility in acrylic pressure-sensitive adhesives (PSAs) and have been associated with reduced cold flow and irritation [8].

Polyethylene Glycol‑Based Acrylate Monomers

Polyethylene glycol (PEG) functional acrylates such as PEG acrylate (PEGA), PEG methacrylate (PEGMA), PEG butyl ether acrylate (PEGBA), PEG octyl ether acrylate (PEGOA), and PEG lauryl ether acrylate (PEGLA) improve water vapor permeability, reduce skin maceration, and can increase patient comfort in long‑wear applications [6].

Polar Functional Monomers for Improved Drug Loading

N‑vinyl lactams (e.g., pyrrolidone derivatives) and hydroxyalkyl monomers such as HEA and HEMA can enhance drug-polymer miscibility and water absorption, thereby supporting higher drug loading or improved release for certain APIs [7,8].

Crosslinking Monomers for Transdermal Drug Delivery System Films and Adhesives

Crosslinking monomers increases mechanical strength, cohesive properties, and patch durability. Trimethylolpropane triacrylate (TMPTA) is widely used to adjust crosslink density and improve mechanical performance [7]. PEG diacrylates and PEG dimethacrylates can form flexible, hydrophilic networks that are useful in drug‑in‑adhesive systems requiring efficient moisture transport [6].

Specialty Monomers for Advanced/Smart Transdermal Drug Delivery Systems

With the emergence of microneedles and stimuli‑responsive patches, specialty monomers have become increasingly important. Biodegradable polyesters derived from lactide and glycolide are used in dissolving microneedles, where monomer selection and polymer architecture govern dissolution kinetics and mechanical strength [9].

**Figure 3:**
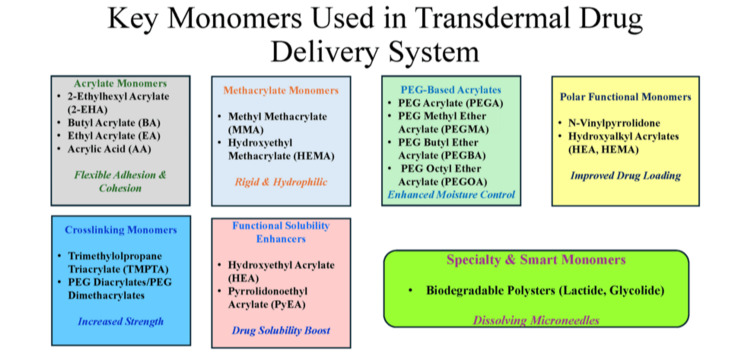
Classification of monomers used in transdermal drug delivery systems and their principal roles. Image credit: Created by the authors using Microsoft PowerPoint (Microsoft Corporation, Redmond, WA, US).

Across formulations, acrylate and methacrylate groups predominate due to their tunable properties and adhesion. PEG acrylates aid with moisture control, while polar and crosslinking monomers enhance solubility, reaction speed, and performance. The final formulation must balance adhesion, cohesion, drug compatibility, skin safety, irritation potential, and regulatory requirements. Figure 3 highlights key monomer categories used in TDDS adhesives and describes how their properties influence adhesion, strength, drug loading, moisture control, and solubility enhancement.

The preceding discussion summarized the principal monomer types utilized in TDDS. Careful selection of monomers is critical to system performance and safety. It determines the chemical and mechanical properties of polymers used in adhesive matrices, backing layers, or rate-controlling membranes. These properties directly influence drug stability, adhesion characteristics, user comfort, and the capacity to regulate drug release [10].

The compatibility between the drug and the polymer is important for patch performance, as monomer selection determines key properties that impact drug solubility, stability, and consistent release [5].

Many TDDS employ acrylic PSAs, which serve as both adhesives and drug reservoirs. Monomers directly influence tack (soft monomers, e.g., 2-ethylhexyl acrylate), cohesion (hard monomers, e.g., MMA or isobornyl methacrylate), and cold-flow behavior (edge ooze). They also affect skin irritation potential and drug-adhesive interactions, which affect flux. Incorporating polar monomers (e.g., HEA) can enhance drug solubility, while macromers may decrease cold flow and irritation [8].

In reservoir-type TDDS, the copolymer composition (monomer ratio or type) influences permeability, mechanical properties, wettability, swelling, and microstructure, all of which affect drug transport. For acrylate copolymers, adjusting monomer ratios can optimize both strength and permeability for different drugs through monomer-driven drug-membrane interactions [11].

Monomers influence hydrolytic and oxidative stability, glass transition temperature (Tg), aging behavior, and long-term chemical stability with drugs and excipients, impacting release kinetics and the shelf life of the drug product [5]. Given multi-day wear, monomer structures affect hypoallergenic properties, irritation potential, moisture vapor transmission, and occlusion, ultimately influencing comfort [8]. Tailored monomers support smart patches, biodegradable matrices, high-drug-load systems, enhanced flexibility, extended wear, and photoreactive crosslinking for UV-curable patches [12-14]. As shown in Table 1, the choice of monomer affects drug-polymer compatibility, adhesion, permeation, mechanical properties, and stability.

**Table 1:** Key performance attributes shaped by monomer choice. Image credit: Created by the authors.

Attribute	Impact of monomer choice
Drug-polymer compatibility	Solubility, stability, crystallization [5]
Adhesion and cohesion	Tack, peel, shear strength, cold flow [8]
Drug permeation rate	Membrane permeability, drug-polymer interactions [11]
Mechanical properties	Flexibility, strength, carrier stability [11]
Skin tolerance	Irritation, comfort, wear time [8]
Stability and aging	Chemical resistance, Tg control, shelf‑life [5]
Advanced patch design	Biodegradability, photo reactivity, custom architectures [12-14]

Polymerization process and impact on performance

Polymerization converts monomers into high-molecular-weight polymers that act as carriers or adhesives. The backbone structure and degree of polymerization affect viscoelasticity, adhesion, and diffusion pathways. Blending polymers can improve release control and reduce skin irritation. Post-polymerization functionalization (e.g., adding polar groups) can enhance release kinetics, encapsulation efficiency, and biocompatibility, but it requires careful regulatory review to ensure safety and stable performance [4,15,16].

Functional groups affect drug-matrix interactions and diffusion mechanisms by altering polarity, hydrogen bonding, and mechanical properties [17]. Hydroxyl‑functional PSAs typically exhibit higher molecular mobility, lower glass transition temperatures, increased tack and viscosity, and weaker drug binding than carboxyl‑functional systems, often resulting in faster release and permeation [18]. In contrast, carboxyl‑functional PSAs can form strong ionic interactions with basic drugs, restrict polymer mobility, and provide comparatively lower permeation; these features are consistent with sustained‑release behavior [18]. Increasing amide content tends to increase hydrogen bonding density and decrease drug release [19]. Methacrylic acid (MAA) and related polar monomers can strengthen specific interactions, reduce release rates, and introduce selectivity in imprinted systems [20]. Beyond functional groups, parameters such as glass transition temperature, free volume, molecular mobility, polarity, hydrophilicity, and thermodynamic activity critically influence drug release [18].

Impact on critical quality attributes

Monomer composition determines the viscoelasticity and the balance of adhesion and cohesion of TDDS adhesives, which, in turn, affect release behavior, stability, and skin compatibility [21]. Consequently, monomer selection is a major factor influencing CQAs, including peel, tack, shear properties, release profile, impurity formation, and skin irritation [22]. Low Tg monomers and long alkyl side chains increase chain mobility and wetting, enhancing tack and initial adhesion. Polar monomers (e.g., AA, HEA) improve wettability and peel strength but may raise irritation or over-adhesion risks at high concentrations. Crosslinkable sites (e.g., carboxyl, epoxide, multifunctional groups) boost shear resistance and reduce cold flow; increased crosslinking improves cohesion, minimizes residue, and preserves structural integrity [22,23]. In acrylic PSAs with different functional groups, guanfacine release and permeation ranked as follows: OH-functional PSA > nonfunctional PSA > COOH-functional PSA. Mechanistically, the trend can be explained by the interaction strength across adhesive types: nonfunctional matrices show almost no binding, OH-containing PSAs exhibit moderate hydrogen bonding because of their lower Tg and higher chain mobility, and COOH-based PSAs display strong ionic pairing [18].

Across different adhesive families and monomer structures, the lidocaine data indicate that acrylate PSAs enable higher drug loading and greater cumulative permeation than silicone or polyisobutylene systems. However, the fraction of drug released does not increase in a simple linear relationship with loading. This shows that compatibility and rheological behavior, defined by the monomer structure, play a greater role in controlling release kinetics than saturation alone [24].

In drug-in-adhesive patches, drug release mainly occurs via simple diffusion, moving from regions of higher to lower concentration within the adhesive. The diffusion rate is strongly influenced by the polymer environment, which is determined by the monomer composition [25]. Increased crosslinking or the incorporation of hydrogen-bond-forming monomers results in a tighter polymer network with reduced free volume, thereby restricting drug mobility and slowing diffusion. Conversely, monomers that increase free volume and enhance polymer chain mobility facilitate drug movement, leading to accelerated diffusion. Thus, adjusting monomer composition offers a practical strategy to control drug-release rates without modifying the drug itself [26].

Residual Monomers and Toxicological Implications

Residual monomers are unavoidable trace impurities remaining after polymerization. Levels vary depending on the process (e.g., approximately 0.01-0.05% for emulsion-polymerized acrylics versus up to 0.1-0.9% for bulk polymerization), creating a potential reservoir of exposure in dermal adhesives [27]. Toxicologically, skin sensitization is the main concern at trace migration levels. MMA is a known weak skin sensitizer with low systemic toxicity; risk assessments show very high safety margins for sensitization induction under conservative scenarios, although elicitation thresholds in sensitized individuals may be lower [28]. Dermatology literature reports acrylate and methacrylate monomers as potent allergens before polymerization; incomplete curing or residual monomers can cause irritant or allergic dermatoses. Strong sensitizers, such as HEMA, triethylene glycol dimethacrylate, and bis-glycidyl methacrylate, support strict residual control for long-wear patches [29]. Emerging data suggest that acrylates are generally more potent sensitizers than methacrylates, warranting tighter limits where possible [30].

Stability Under Stress (Heat, Humidity)

Higher levels of “soft” monomers (e.g., 2‑EHA, BA) lower Tg and increase drug mobility, boosting tack at room temperature but increasing susceptibility to flow, deformation, and loss of cohesion at elevated temperatures. UV‑crosslinked acrylic copolymers with BA/2‑EHA/AA show that monomer identity and crosslink density significantly affect shear strength at 20°C and 70°C [31]. Free volume expansion and chain mobility increase with temperature and humidity; monomer-driven Tg and crosslink density govern these responses, linking the same structural variables that determine diffusion to thermomechanical stability (creep, cold flow, delamination) [32].

Humidity-driven adhesive shrinkage and deformation have also been linked to monomer composition. Studies of acrylic PSAs with varying residual monomer content showed that formulations containing less residual monomer offered better mechanical stability, including less shrinkage, while higher monomer levels increased instability. Although not specific to TDDS, this evidence directly applies to pharmaceutical PSAs, as residual monomer contributes to plasticization and accelerates instability under heat and moisture exposure. In summary, achieving an optimal balance between soft and hard monomer characteristics, together with appropriate crosslinking, improves the robustness of TDDS under thermal and humidity stress [33].

Regulatory considerations

Food and Drug Administration and European Medicines Agency Expectations for Monomer Characterization

Food and Drug Administration (FDA) guidance highlights the importance of fully characterizing the adhesive composition because it affects product design, performance, and safety. A well-defined adhesive system helps reduce the risk of adhesion failure, supports consistent dose delivery, and protects patient safety. The selection of monomers, including choices between soft and hard monomers and the use of cross-linkable species, is considered a critical material attribute and requires scientific justification [10]. The European Medicines Agency (EMA) quality guideline for transdermal patches also requires complete disclosure and justification of the adhesive formulation. This includes the type of adhesive polymer and any monomer-derived excipients. The characteristics of the monomers, such as polarity, functional groups, and molecular weight, are viewed as key factors that define the CQAs of the final product [34].

Common expectations include complete identification and justification of the adhesive monomer composition, quantitative control of residual monomers within extractables and leachables (E&L)/impurity specifications, assessment of adhesive performance (adhesion, stability, rheology), toxicological risk assessment (notably sensitization), and demonstrated CQA impact (release, adhesion/cohesion, stress stability).

Residual Monomer Limits and Toxicology Assessments

The FDA does not set universal ppm limits for all residual monomers in TDDS; limits are specific to each compound and based on risk assessments (such as sensitization, systemic toxicity, migration, and clinical use). Related sectors aim to reduce hazardous monomers to the lowest technically achievable level (e.g., acrylamide ≤5 ppm in certain cosmetics) [35]. FDA/Product Quality Research Institute promotes an E&L-driven approach, utilizing appropriate analytics (often gas chromatography-flame ionization detection (GC-FID)/gas chromatography-mass spectrometry (GC-MS)) and conservative dermal exposure toxicological risk assessment; incomplete E&L data can delay approval processes [36]. EMA considers residual monomers as process-related impurities of the excipient that require strict control and analytical verification, in line with European Chemicals Agency guidance on unreacted monomers in polymers [34,37]. The toxicology focus for acrylates and methacrylates centers on skin sensitization; formal evaluations for MMA indicate high safety margins for induction, with particular attention to elicitation in sensitized individuals [38,39].

Analytical and characterization techniques

Residual Monomer Quantification

GC-FID and GC-MS are primary methods for detecting volatile and semi-volatile monomers at trace levels; GC-MS enhances specificity when matrix interference is significant. Validated multi-analyte GC-MS methods can measure multiple monomers simultaneously in complex adhesives using optimized extraction and matrix-matched calibration [40]. For non-volatile or thermally labile monomers, use HPLC or derivatization-assisted GC; 1H/13C nuclear magnetic resonance can validate GC data and directly confirm residuals in crosslinked systems. These approaches are consistent with E&L expectations for quantitative identification [41].

Polymer Characterization

Fourier transform infrared spectroscopy (FTIR) and Raman confirm functional groups, monomer incorporation, and API dispersion. Raman microscopy is advantageous because it can distinguish between dissolved and suspended APIs, monitor recrystallization, and distinguish among chemically similar acrylics [42]. Differential scanning calorimetry provides Tg and thermal behavior, which depend on the monomer and the degree of crosslinking. Scanning electron microscopy reveals microstructure and morphological changes caused by monomer leaching or recrystallization [41]. Viscoelastic and mechanical profiling (dynamic mechanical analysis, tensile tests, fracture energy) measures how monomer ratios and architecture influence adhesion and cohesion; viscoelastic window analysis and double cantilever beam fracture testing are illustrative [43]. FTIR-ATR and molecular dynamics simulations connect monomer-driven mobility, free volume, and crosslink density to drug diffusion in PSAs [26].

Challenges and emerging trends

Monomer Migration and Patch Integrity

Residual or incompletely polymerized species may be more mobile, especially when exposed to heat, humidity, and occlusion. Free volume and segmental mobility correlate positively with diffusion, while hydrogen bonding and ionic interactions hinder it. These factors influence safety and mechanical performance, such as adhesion, cohesion, and dimensional stability. Crosslink density, substrate roughness, stacked layers, and PSA thickness all affect tensile strength, fracture energy, and overall performance [4].

Innovations in Monomer Design

Varying functional monomers (hydroxyl, carboxyl, amide) allows precise control of drug miscibility, mobility, and release. Hydrogen-bond donor/acceptor monomers regulate the strength of drug-polymer association, enabling tuning of flux, lag time, and release linearity [39]. These supports extend TDDS to hydrophilic and amphiphilic drugs via engineered microenvironments.

Regulatory gaps and future directions

Although FDA and EMA guidelines recommend full characterization of PSAs, they do not specify explicit monomer-specific quantitative limits for use in TDDS. The agencies instead rely on risk-based E&L approaches and general impurity principles, leaving a gap for monomer-specific toxicological thresholds. Regulators require demonstration of safe monomer exposure through migration studies and toxicology risk assessments, but the lack of standardized monomer acceptance criteria leads to variability among manufacturers [23]. Regulatory systems increasingly favor in vitro study data to demonstrate product quality and bioequivalence. FDA and EMA guidelines highlight that in vitro release testing and in vitro permeation testing data, combined with physicochemical and structural equivalence, may reduce clinical study requirements. However, current guidance does not fully incorporate monomer migration effects into regulatory expectations for in vitro studies, despite evidence that monomer chemistry influences drug release [36,44]. Unlike injectables or inhalation products, topical/transdermal E&L frameworks are less standardized. Current gaps include the lack of unified global standards for residual monomer quantification thresholds, inconsistent expectations for stress condition migration testing involving heat and humidity, and limited regulatory guidance on monomer-driven patch integrity failures (e.g., adhesion drift due to monomer plasticization).

As polymer science progresses, there is increasing recognition, supported by mechanistic PSA and patch microstructure studies, that monomer-driven changes in viscoelasticity and drug-polymer affinity should be more explicitly incorporated into regulatory decision frameworks [4,45].

Based on current literature and regulatory momentum, future directions likely include standardized monomer-specific reporting requirements in Module 3, stricter monomer migration E&L acceptance criteria for long-wearing patches, integration of predictive polymer modeling (e.g., MD simulations validated in PSA studies) into regulatory science [39], and expansion of EMA/FDA frameworks aimed to include adhesive chemistry effects, including monomer-dependent release behavior, explicitly [24,44].

Recommendations

Effective selection of monomers for transdermal systems often benefits from a structured approach that begins with thorough monomer characterization, as early clarity often supports later decisions on formulation performance, safety, and regulatory expectations. Alignment between monomer functionality and the properties of the drug substance also contributes to more predictable release behavior, whether through enhanced mobility for hydrophilic drugs or stronger interactions for drugs requiring controlled release. Careful attention to residual monomer levels remains important to ensure both product quality and patient safety, particularly when supported by sensitive, validated analytical methods. The ratio of soft to hard monomers typically influences the adhesive’s viscoelastic balance, thereby affecting adhesion, cold flow, and overall patch integrity under environmental stresses. Within Quality by Design frameworks, monomer composition is often a critical material attribute that shapes key quality attributes, including adhesion, cohesion, drug release, and stability. Broader safety and regulatory considerations continue to reflect the importance of monomer-specific E&L assessments, toxicological evaluations, and harmonized expectations for reporting and residual limits. Advances in analytical science and computational modeling, including spectroscopic, microscopic, and simulation-based tools, provide deeper insight into monomer-driven polymer behavior and support more informed formulation strategies.

## Conclusions

Monomers form the molecular foundation of TDDS polymers and adhesives, and their selection significantly affects product quality, efficacy, and safety. The chemical structure, polarity, and functional groups of monomers determine polymer mobility, drug compatibility, adhesion, cohesion, membrane permeability, and both mechanical and thermal stability. Functional monomers, including those with carboxyl, hydroxyl, or amide groups, modulate drug-polymer interactions and release kinetics. In contrast, the ratio of soft to hard monomers governs tack, cohesion, and wear properties. Residual monomers represent a critical safety issue due to their potential to cause skin sensitization, necessitating rigorous analytical controls and comprehensive toxicological evaluation. Regulatory agencies (FDA/EMA) require detailed monomer characterization, justification of adhesive properties, and strict control of residual monomers within E&L programs. As TDDS evolve to accommodate higher drug loads, longer wear times, and advanced functionalities, precise monomer engineering becomes increasingly important for achieving optimal performance, regulatory compliance, and patient acceptance.
